# Clinical diagnosis of TB: lessons on misdiagnosis and overdiagnosis

**DOI:** 10.5588/pha.25.0012

**Published:** 2025-06-04

**Authors:** D.S. Singini, N. Sanjase, M. Kagujje, J. Shatalimi, C.P. Chisanga, Z.D. Lupatali, D. Phiri, T. Tatila, W. Olwit, A.D. Kerkhoff, M. Muyoyeta

**Affiliations:** ^1^ Centre for Infectious Disease Research in Zambia, Lusaka, Zambia;; ^2^ The Ministry of Health, Lusaka, Zambia;; ^3^ Uganda Cancer Institute, Kampala, Uganda;; ^4^ University of California, Division of HIV, Infectious Diseases, and Global Medicine, Department of Medicine, Zuckerberg San Francisco General Hospital, San Francisco, USA.

**Keywords:** tuberculosis, chest X-ray, unconfirmed TB, computer-aided diagnosis, TB burden, Zambia

## Abstract

Clinically diagnosed TB patients (n = 335) at two facilities in Lusaka, Zambia were re-evaluated within two weeks of diagnosis. This re-evaluation included sputum Xpert Ultra testing and expert reader interpretation of the chest x-rays (CXRs) used for initial diagnosis. Repeat Xpert Ultra detected TB in just 2.6% (n=6). Of the remaining patients (n=222), expert CXR re-interpretation classified 18.0% as normal; 36.0% as abnormal, consistent with TB; and 46.0% as abnormal, not consistent with TB. These findings suggest that clinical TB is frequently over diagnosed in those without detectable CXR abnormalities and misdiagnosed in those with abnormal CXRs: these abnormalities are likely due to other respiratory conditions. Such misdiagnosis leads to unnecessary treatment, failure to treat the true underlying condition and incorrect estimates of TB burden.

Over the past decade, there has been a substantial increase in access to rapid and sensitive TB diagnostic tools, yet bacteriological confirmation of TB at diagnosis remains a key challenge in many high burden settings.^1^ Bacteriological confirmation is critical for correct diagnosis and resistance detection, but in 2022, only 63% of global TB notifications were bacteriologically confirmed.^2^ The lowest rates of bacteriological confirmation are in high-burden, low-income countries (LMICs), with many countries reporting over 50% of TB notifications without bacteriological confirmation.^2^ This is largely due to limited access to sensitive diagnostic tools and, where these are available, delays in receiving results.^3^ Furthermore, post-COVID-19, pressure to increase TB case detection to pre-pandemic levels, combined with national TB programs’ urgency to reduce the incidence of TB and mortality, may have also contributed to increased clinical diagnosis in recent years.^4^ In Zambia, despite improved access to Xpert Ultra, the proportion of clinically diagnosed TB has remained consistently high – i.e., 43% in 2023 compared to 44% in 2018.^2^ In this study, we sought to determine the extent of potential misdiagnosis and overdiagnosis among clinically diagnosed TB patients.

## METHODS

During the implementation of the USAID TBLON project, we conducted a cross-sectional study in which patients with a clinical diagnosis of TB (n = 335) were prospectively enrolled at 2 health facilities within Lusaka urban district, between December 2021 to July 2022. These facilities were purposefully selected because of the high proportion (above 55%) of clinically diagnosed TB patients relative to the total notifications at the facilities. Individuals ≥15 years old, who had been clinically diagnosed with pulmonary TB and had been on treatment for less than 2 weeks were eligible for inclusion. At enrolment, participants were subjected to repeat sputum examination using Xpert Ultra, and chest X-ray (CXR) review by a highly experienced radiologist**.** Baseline demographics and study-related data were extracted from the TB treatment register and from participants and then entered into the District Health Information System 2 (DHIS2) under the tracker capture application. Data was analysed using Stata version 18.0. Chi-squared tests or Fisher’s exact tests were used as appropriate to compare proportions, whereas Wilcoxon rank-sum or Kruskal-Wallis tests were used to compare medians as appropriate.

All participants provided written informed consent. The study received ethical approval from the University of Zambia biomedical research ethics committee (ref: 1773-2021).

## RESULTS

Of 335 participants with a clinical TB diagnosis enrolled into the study, 103 (30.7%) had missing CXRs and 4 (1.2%) had poor quality CXRs that were uninterpretable (Figure). Therefore, 228 (68.1%) participants had CXRs available that were evaluated by an expert reader. Among these, the median age was 31 (IQR 29–47) years, 73.6% were male, 41.7% reported being HIV positive, and 25.4% reported a prior TB treatment history (Table). All had a positive WHO symptom screen with cough being almost universally present.

**FIGURE.**
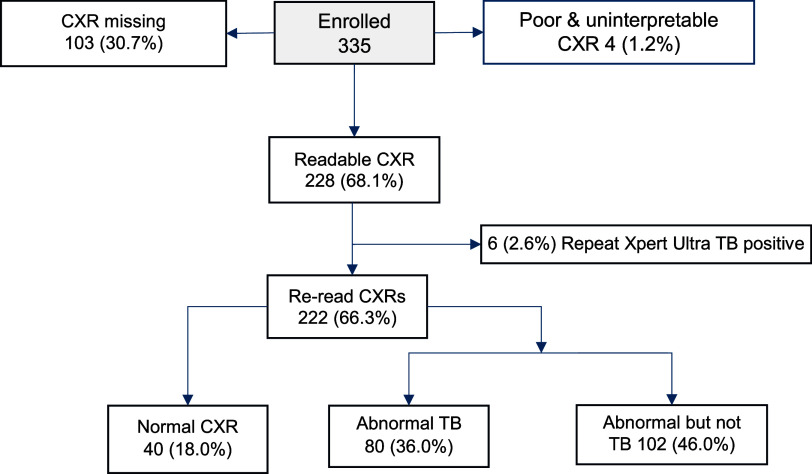
Study flow diagram.

**TABLE.** Baseline characteristics of participants with CXRs available for evaluation by an expert reader.

Characteristics	Total number of participants (N = 228)
Median age (IQR)	38 (29–47)
Sex	
Male	166 (72.8%)
Female	57 (35.0%)
HIV Status	
Positive	95 (41.7%)
Negative	133 (58.3%)
TB patient type	
New	170 (74.6%)
Previously treated	58 (25.4%)
Symptoms	
WHO screen positive	228 (100%)
Cough	215 (94.3%)
Weight loss	190 (83.3%)
Night sweats	179 (78.5%)
Fever	69 (30.3%)

Repeat Xpert Ultra testing detected TB in only 6 participants (2.6%). Among the remaining 222 participants without microbiological evidence of TB, 40 (18.0%) had a normal appearing CXR, 80 (36.0%) had an abnormal CXR consistent with TB, and 102 (46.0%) had an abnormal CXR, not consistent with TB (see Figure).

## DISCUSSION

Making a clinical diagnosis of TB can be challenging due to difficulties in distinguishing TB from other illnesses with similar clinical presentations.^5^ Our results provide evidence of the substantial risk of misdiagnosis and overdiagnosis of clinical TB in Zambia. In such high TB burden and low-resource settings, when confirmatory tests are negative the reliance on CXR is high. Although such settings have populations at high risk of other respiratory diseases that may mimic TB, such as silicosis, pneumonia, chronic obstructive pulmonary disease (COPD) and post TB lung disease (PTLD), the index of suspicion for these conditions is low due to the lack of diagnostic tools and their unknown burden.^6,7^ The limited access to expert CXR readers also likely contributes to this misdiagnosis of clinical TB.^8^ The challenge of accurately interpreting CXRs is complicated by the atypical presentation of TB in people living with HIV (PLHIV) and the significant overlap in CXR patterns between TB and several of these other respiratory conditions.^9^ This was evident in our study, where nearly half of clinically diagnosed TB patients had CXRs that the expert radiologist classified as abnormal, but not consistent with TB. Particularly concerning was the clinical diagnosis of TB in the almost 20% of patients with CXRs interpreted as not having any detectable abnormalities (i.e., normal) or poor quality. Such misdiagnosis risks unnecessary exposure to treatment and side effects, increased antimicrobial resistance (AMR) and incorrect estimates for TB burden and case detection rates.

Limitations to our study include the small sample size, a relatively high proportion of missing CXRs, and limited medical history and physical examination that could aid in making alternative diagnoses. Despite these limitations, our study provides important insights from a real-world setting into the high frequency of misdiagnosis when a clinical TB diagnosis is made.

In conclusion, these findings underscore the need for capacity building to develop a healthcare workforce skilled in CXR interpretation and for comprehensive approaches to screening and diagnosing TB and other lung conditions. In low-resource settings, where expert readers are unavailable, computer-aided diagnosis (CAD) systems could improve clinical TB diagnosis.^10^ Misdiagnosis of clinical TB risks incorrect estimates of TB burden and case detection rates. Larger studies, with a comprehensive evaluation and alternative diagnosis, are required to verify our findings.

## References

[1] Nema, V., Tuberculosis diagnostics: Challenges and opportunities. Lung India, 2012;29(3):259-66. 22919166 10.4103/0970-2113.99112 PMC3424866

[2] World Health Organization. Global Tuberculosis reports 2019-2024. Geneva, Switzerland: WHO.

[3] Vasiliu, A., , Implementing molecular tuberculosis diagnostic methods in limited-resource and high-burden countries. Breathe (Sheff), 2022;18(4):220226. 36865933 10.1183/20734735.0226-2022 PMC9973455

[4] World Health Organization. Implementing the end TB strategy: the essentials, 2022 update. Geneva, Switzerland: WHO, 2022.

[5] Davies, P.D. and M. Pai, The diagnosis and misdiagnosis of tuberculosis. Int J Tuberc Lung Dis, 2008;12(11):1226-34. 18926032

[6] Ekeng, B.E., , Pulmonary and Extrapulmonary Manifestations of Fungal Infections Misdiagnosed as Tuberculosis: The Need for Prompt Diagnosis and Management. J Fungi (Basel), 2022;8(5):doi.org/10.3390/jof8050460. PMC9143176 35628715

[7] Visca, D., , Post-TB disease: a new topic for investigation-and why it matters. Int J Tuberc Lung Dis, 2021;25(4):258-261. 33762068 10.5588/ijtld.21.0040

[8] Meghji, J., , A Systematic Review of the Prevalence and Pattern of Imaging Defined Post-TB Lung Disease. PLoS One, 2016;11(8):e0161176. 27518438 10.1371/journal.pone.0161176 PMC4982669

[9] Mateyo, K., , Clinical and radiographic characteristics of presumptive tuberculosis patients previously treated for tuberculosis in Zambia. PLoS One 2022;17(1):e0263116. 35085353 10.1371/journal.pone.0263116 PMC8794156

[10] World Health Organization. WHO Consolidated guidelines on tuberculosis: module 2: screening: systematic screening for tuberculosis disease, in Module 2: Screening. Geneva, Switzerland: WHO, 2021. 33822560

